# Comparison of upstream regulators in human *ex vivo* cultured cornea limbal epithelial stem cells and differentiated corneal epithelial cells

**DOI:** 10.1186/1471-2164-14-900

**Published:** 2013-12-17

**Authors:** Zoltán Veréb, Réka Albert, Szilárd Póliska, Ole Kristoffer Olstad, Saeed Akhtar, Morten C Moe, Goran Petrovski

**Affiliations:** 1 Stem Cells and Eye Research Laboratory, University of Debrecen, Debrecen, Hungary; 2 Center for Clinical Genomics and Personalized Medicine, Department of Biochemistry and Molecular Biology, Medical and Health Science Center, University of Debrecen, Debrecen, Hungary; 3 Department of Medical Biochemistry, Oslo University Hospital and University of Oslo, Oslo, Norway; 4 Cornea Research Chair, Department of Optometry, College of Applied Medical Sciences, King Saud University, Riyadh, Saudi Arabia; 5 Centre of Eye Research, Department of Ophthalmology, Oslo University Hospital, University of Oslo, Oslo, Norway; 6 Apoptosis and Genomics Research Group of Hungarian Academy of Sciences, Medical and Health Science Center, University of Debrecen, Debrecen, Hungary; 7 Department of Ophthalmology, Faculty of Medicine, University of Szeged, Korányi fasor 10-11, 6720, Szeged, Hungary

**Keywords:** Limbal epithelial stem cells, Corneal epithelial cells, Gene array, Upstream regulators, Cytokines, Cell adhesion, Angiogenesis, IL-6, IL-8

## Abstract

**Background:**

The surface of the human eye is covered by corneal epithelial cells (CECs) which regenerate from a small population of limbal epithelial stem cells (LESCs). Cell therapy with LESCs is a non-penetrating treatment for preventing blindness due to LESC deficiency or dysfunction. Our aim was to identify new putative molecular markers and upstream regulators in the LESCs and associated molecular pathways.

**Results:**

Genome-wide microarray transcriptional profiling was used to compare LESCs to differentiated human CECs. Ingenuity-based pathway analysis was applied to identify upstream regulators and pathways specific to LESCs. ELISA and flow cytometry were used to measure secreted and surface expressed proteins, respectively. More than 2 fold increase and decrease in expression could be found in 1830 genes between the two cell types. A number of molecules functioning in cellular movement (381), proliferation (567), development (552), death and survival (520), and cell-to-cell signaling (290) were detected having top biological functions in LESCs and several of these were confirmed by flow cytometric surface protein analysis. Custom-selected gene groups related to stemness, differentiation, cell adhesion, cytokines and growth factors as well as angiogenesis could be analyzed. The results show that LESCs play a key role not only in epithelial differentiation and tissue repair, but also in controlling angiogenesis and extracellular matrix integrity. Some pro-inflammatory cytokines were found to be important in stemness-, differentiation- and angiogenesis-related biological functions: IL-6 and IL-8 participated in most of these biological pathways as validated by their secretion from LESC cultures.

**Conclusions:**

The gene and molecular pathways may provide a more specific understanding of the signaling molecules associated with LESCs, therefore, help better identify and use these cells in the treatment of ocular surface diseases.

## Background

The cornea serves mainly a protective and refractive function, being found on the outermost surface of the eye. It is a highly transparent and strong tissue, separated from the surrounding conjunctiva by a transitional zone - the limbus. During eye development, the cornea is the last part of the eye to be formed. It consists of a stratified epithelium at the surface, stroma in the middle - populated by keratocytes and fibroblast-like cells, and an inner layer of endothelial cells, each separated by a Bowman’s and Descemet’s membrane, respectively.

The human central corneal epithelial cells (CECs) are derived and replaced continuously from the limbal epithelial stem cells (LESC). The later can undergo asymmetric division and give rise to transient amplifying cells (TACs), which can then differentiate into mature CECs that lose their ability to proliferate [1,2]. Animal studies have shown that CECs arise from approximately 100 progenitor cells, which means the frequency of LESCs is extremely low [3]. In humans, the LESCs have been found in the limbal epithelial crypts - special niches at the peripheral edge of the cornea [4-6]. Only six such crypts have been identified in the limbus, further strengthened by findings from animals [4]. The crypts provide a concentrated and safe place for harboring LESCs, and also, a rich vascular supply with growth factors and metabolites for their sustained persistence [1,7-10]. LESCs play a key role not only in epithelial differentiation, but also in wound healing, tissue regeneration and maintenance of a balanced immunological state in the cornea [11].

Injuries - traumatic, chemical or iatrogenic, or diseases of the LESCs, either inborn or acquired, can all lead to partial or total LESC deficiency (LESCD) or corneal neovascularization accompanied by inflammation. Full penetrating keratoplasty is not anymore the mainstay of treatment for LESCDs, while autologous limbal graft transplantation from a healthy donor eye, if available, does not provide a guarantee for the functionality of the graft itself. Isolation and *ex vivo* expansion of autologous or homologous LESCs in human-like conditions has only been described in detail in the last couple of years [12]. We recently published a method for cultivating and characterizing LESCs grown on lens capsule in a medium containing human serum as the only growth supplement [13]. The benefit of our method is not only the use of animal material-free culturing conditions, but also, the ability to investigate the phenotype and the genotype of the outgrowing cells, which can further help identify new putative LESC markers.

In the present study, we compare the gene expression patterns of *ex vivo* cultured human LESCs to differentiated CECs with a main focus on markers for stemness and proliferation, epithelial differentiation, tissue development and growth, immunological and angiogenic factors. In addition, we propose a way to identify and possibly concentrate these stem cells found at low density from the heterogeneous cell populations found in the cornea for future use in clinical transplantation.

## Methods

### Ethics statement

All tissue collection complied with the guidelines of the Helsinki Declaration and was approved by the Regional Ethical Committee (DEOEC RKEB/IKEB 3094/2010). Limbal tissue collection was done within 12 hours of biologic death from cadavers only and Hungary follows the EU Member States’ Directive 2004/23/EC on presumed consent practice for tissue collection [14].

### Isolation and cultivation of LESCs and CECs

In brief, after a thorough eye wash with 5% povidone iodine (Betadine; Egis Pharmaceuticals PLC, Budapest Hungary), the conjunctiva was incised and separated from the limbal junction; consequently, a 2 × 1 mm rectangular-shaped limbal graft was dissected away and towards the cornea, respectively, at the 12 o’clock position. The depth of the graft was kept superficial or within the epithelial layer; multiple grafts were collected from a single eye and tested for growth potential. The graft dissection was performed using a lamellar knife placed tangential to the surface being cut. LESCs were cultured in a high-glucose Dulbecco-modified Eagle’s medium (DMEM-HG, Sigma-Aldrich, Budapest, Hungary) supplemented with 20% v/v human AB serum, 200 mM/mL L-glutamine, 10,000 U/mL penicillin- 10 mg/mL streptomycin (all from Sigma-Aldrich) at 37°C, 5% CO_2_ in 1.91 cm^2^ tissue culture plates, while the medium was changed every alternate day. The growth of the cells was monitored under phase contrast microscope regularly. Only grafts which had cell outgrowth within 24 hours were processed further to decrease the chance of fibroblast contamination and maintained in culture up to 14 days when they reached 95-100% confluence. Differentiated CECs were scraped from the central part of the cornea of cadavers and were used as a positive control. To avoid contamination of one or the other cell type during isolation, different donors were used for each isolation carried out.

### Microarray and data analysis

Affymetrix GeneChip Human Gene 1.0 ST Arrays (Affymetrix, Santa Clara, CA, USA) were used for the microarray analysis. The array contained more than 28,000 gene transcripts. For the whole genome gene expression analysis 150 ng of total RNA was subjected to Ambion WT Expression Kit (Ambion, Life Technologies, Carlsbad, CA, USA) and GeneChip WT Terminal Labeling Kit (Affymetrix) according to the manufacturers’ protocols. After washing, the arrays were stained using the FS-450 fluidics station (Affymetrix) and signal intensities were detected by Hewlett Packard Gene Array Scanner 3000 7G (Hewlett Packard, Palo Alto, CA, USA). The scanned images were processed using GeneChip Command Console Software (AGCC) (Affymetrix) and the CEL files were imported into Partek Genomics Suite software (Partek, Inc. MO, USA). Robust microarray analysis (RMA) was applied for normalization. Gene transcripts with a maximal signal values less than 32 across all arrays were removed to filter for low and non-expressed genes, reducing the number of gene transcripts to 23190. Differentially expressed genes between groups were identified using one-way ANOVA analysis in Partek Genomics Suite Software. Clustering analysis was made using the same name module in a Partek Genomics Suite Software.

### Pathway analysis

To identify the relationships between selected genes, the Ingenuity Pathway Analysis (IPA, Ingenuity Systems, Redwood City, CA) was used. Excel datasheets containing gene IDs with the assigned gene expression values were uploaded into the program. The Ingenuity Pathways Knowledge Base (IPKB) provided all known functions and interactions which were published in the literature. ANOVA was used to calculate a p-value to determine the probability that each biologic function or canonical pathway assigned to the data set was due to chance alone. For the representation of the relationships between the genes, the ‘Pathway Designer’ tool of the IPA software was used.

### Measurement of cytokine concentrations by ELISA

LESCs growing out of the limbal grafts were trypsinized (0.025% trypsin-EDTA (PAA, Pasching, Austria, 5 minutes, 37°C) and seeded onto 24-well plates at a 5×10^4^ cell/mL density. Cells were cultured for 9 to 13 days. At the end of the culturing period, the supernatants were harvested and kept at −20°C until further measurement. BD OptEIA ELISA (BD Pharmingen, San Diego, CA, USA) assay kits were used following the supplier’s instruction to measure the concentration of secreted IL-6 and IL-8 cytokines. Each experiment was performed at least three times and each sample was tested in triplicates. Statistically significant differences were determined by paired student’s t test.

### Transmission electron microscopy

Human corneal tissue procurement and use were conducted in accordance with local regulations and approved by the Research Ethics Committee of King Saud University. Unless specified otherwise, reagents were obtained from TAAB Laboratories Equipment Ltd (Aldermaston, UK). Pieces of LESCs grown on lens capsules were fixed in freshly prepared 4% paraformaldehyde in 0.1 M phosphate for 2 h at 4°C. Tissues were processed at low temperatures and were embedded in LR White resin (Sigma-Aldrich) at −20°C for 48 h under ultraviolet light. Ultrathin sections were collected on 200 mesh formvar-coated carbon nickel grids and examined in a Jeol 1400 transmission electron microscope (Jeol Ltd, Tokyo, Japan).

### Surface protein level analysis by flow cytometry

Fluorescein isothiocyanate (FITC), phycoerythrin (PE) and allophycocyanin (APC) conjugated antibodies were used for multicolour flow cytometric analysis to measure the selected surface protein expression on isolated LESCs and differentiated CECs. Antibodies against CD29/Integrin β1, CD44/HCAM, CD45, CD54/ICAM1, CD73, CD90/Thy-1, CD117/c-kit and CD146/MCAM markers were used in a concentration specified by the manufacturer’s protocol (all antibodies were obtained from Biolegend, San Diego, CA, USA). All samples were labeled for 30 minutes on ice, then measured by FACSCalibur flow cytometer (BD Biosciences Immunocytometry Systems, San Jose, CA, USA) and the data were analyzed using FlowJo (TreeStar, Ashland, OR, USA), software. The results were expressed as means of positive cells (%) *±* SD. Statistically significant difference between the two groups (LESC vs. CECS) was determined with paired student-t test and a value of p < 0.05 was considered significant.

## Results

### Gene array and IPA analysis

A microarray based transcriptional profiling was used to compare LESCs to differentiated CECs. The intensity profiles of the log_2_ transformed signal values of the 28869 transcripts were obtained, out of which 955 and 875 transcripts had a more than 2 fold change (FC) increase and decrease in expression between the two cell types, respectively (*n* = 3, *p* < 0.01). Table 1 summarizes the most affected signaling pathways identified by the IPA software based on the significant expression of genes in the LESCs. The top canonical pathways included genes involved in hepatic fibrosis, angiogenesis inhibition by thrombospondin 1 (TSP-1), retinoic acid receptor (RAR) activation, antigen presentation and axonal guidance signaling. Some of the signaling pathways were also related to diseases or toxicological pathways such as induction of reactive metabolites, renal ischemia and renal proliferation. IPA could determine the biological functions and diseases from the significantly changed expression levels of groups of genes: 733 molecules were found to be involved in cancer development, 567 in cellular growth and proliferation, 552 in cellular development, 520 in cell death and survival and 402 in gastrointestinal diseases. Only a small number of molecules related to visual system development and function (98), and 5 involved in increased levels of albumin could be detected. (for more details see Table 2).

**Table 1:** The most significantly affected canonical pathways found in LESCs

	**Ingenuity canonical pathways**	**-Log(B-H-P-value)**	**Ratio**
**Top canonical pathways**	Hepatic fibrosis/hepatic stellate cell activation	8.36E-05	32/142 (0.225)
	Inhibition of angiogenesis by TSP1	2.22E-04	12/34 (0.353)
	RAR activation	5.01E-04	35/179 (0.196)
	Antigen presentation pathway	1.25E-03	11/40 (0.275)
	Axonal guidance signaling	1.31E-03	65/432 (0.15)
**Top tox (toxicological) pathways**	Hepatic fibrosis	4.25E-06	27/93 (0.29)
	Glutathione depletion - cyp induction and reactive metabolites	6.6E-05	7/12 (0.583)
	Liver proliferation	1.5E-04	39/189 (0.206)
	Persistent renal ischemia-Reperfusion injury (mouse)	4.41E-04	10/30 (0.333)
	Increases renal proliferation	4.54E-04	24/101 (0.238)

IPA software was used to calculate the canonical pathways from the gene expression profile of LESCs grown on lens capsule.

**Table 2:** Top biological and toxicological functions

**Function**	**Name**	**p value**	**Molecules**
**Diseases and disorders**	Cancer	5.96E-27 - 1.23E-03	733
	Reproductive system disease	1.61E-16 - 1.19E-03	344
	Dermatological diseases and conditions	3.42E-16 - 1.15E-03	282
	Gastrointestinal disease	4.31E-13 - 8.26E-04	402
	Endocrine system disorders	2.65E-10 - 7.46E-04	257
**Molecular and cellular functions**	Cellular movement	5.90E-18 - 1.43E-03	381
	Cellular growth and proliferation	1.31E-10 - 1.12E-03	567
	Cellular development	3.26E-09 - 1.07E-03	552
	Cell-to-cell signaling and interaction	8.23E-09 - 1.48E-03	290
	Cell death and survival	1.04E-08 - 1.48E-03	520
**Physiological system development and function**	Cardiovascular system development and function	9.52E-10 - 1.23E-03	271
	Tumor morphology	6.48E-09 - 9.47E-04	140
	Organismal development	9.59E-09 - 1.48E-03	371
	Visual system development and function	1.34E-07 - 1.48E-03	98
	Tissue development	2.59E-07 - 1.48E-03	350
**Clinical chemistry and hematology**	Decreased levels of albumin	1.63E-03 - 3.94E-01	6
	Increased levels of alkaline phosphatase	2.79E-03 - 1.18E-01	16
	Increased levels of creatinine	8.01E-03 - 8.01E-03	8
	Increased levels of potassium	1.48E-02 - 5.41E-01	7
	Increased levels of albumin	1.09E-01 - 2.21E-01	5
**Cardiotoxicity**	Cardiac stenosis	6.92E-04 - 3.13E-01	15
	Congenital heart anomaly	3.64E-03 - 5.28E-01	23
	Cardiac arteriopathy	4.20E-03 - 6.33E-01	42
	Pulmonary hypertension	5.95E-03 - 1.18E-01	11
	Cardiac hypertrophy	8.04E-03 - 1.00E00	51
**Hepatotoxicity**	Liver proliferation	2.37E-04 - 3.13E-01	39
	Liver cholestasis	6.93E-04 - 5.84E-01	22
	Liver cirrhosis	7.33E-04 - 2.21E-01	31
	Liver damage	8.26E-04 - 2.21E-01	33
	Liver hyperplasia/hyperproliferation	8.39E-03 - 5.03E-01	80
**Nephrotoxicity**	Renal proliferation	6.16E-06 - 2.21E-01	38
	Renal damage	7.17E-04 - 5.03E-01	37
	Renal tubule injury	7.17E-04 - 2.21E-01	24
	Renal necrosis/cell death	1.84E-03 - 1.00E00	52
	Renal inflammation	8.70E-03 - 1.00E00	34

The most affected pathways related to known biological and toxicological functions in LESCs as determined by the IPA software.

### Customized gene array data – upstream regulators

**Figure 1:**
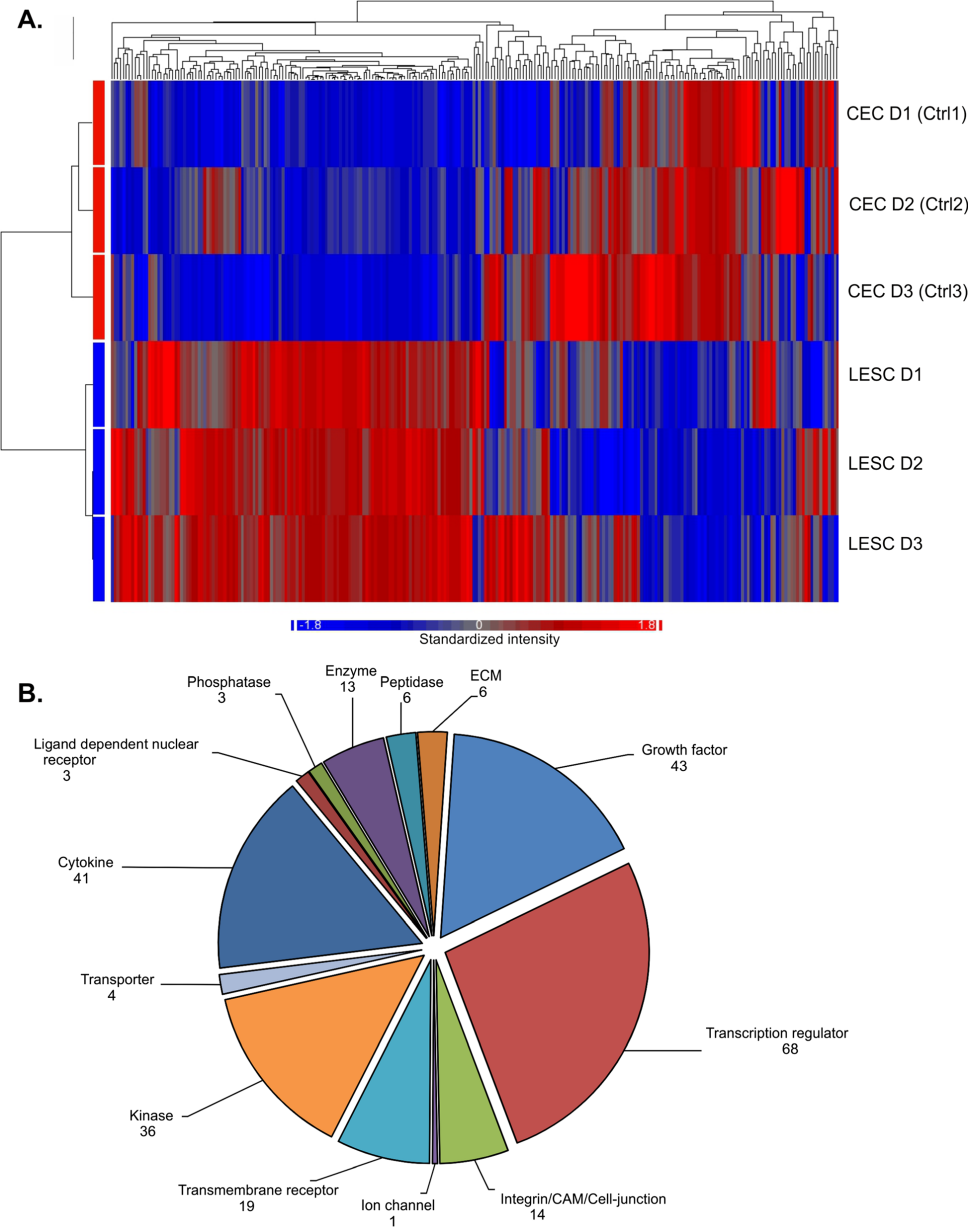
**Heatmap of the differentially expressed genes in LESCs compared to CECs.** Heatmap of the transcripts and functional clustering of 257 genes expressed significantly different in LESCs and CECs, and related to stemness, epithelial differentiation, tissue organization and angiogenesis. Red and blue colors indicate high and low expression, respectively. The cluster analysis and dendrogram show the difference between the two cell types **(A)**. Distribution of the 257 significantly differentially expressed genes by molecule type as defined by IPA **(B)**.

We selected 257 upstream regulators that were expressed significantly and differentially in LESCs that were also related to our groups of interest (stemness and proliferation, differentiation, cell adhesion, cytokines and growth factors and angiogenesis). Their biological functions were extensively related to physiological maintenance of LESCs, while the molecules involved in these processes showed significant inter-donor differences. Figure 1 shows the heatmap and the functional clustering of the 257 upstream regulators selected on the basis of their high or low FC or previously documented relation to LESCs. The cluster analysis demonstrated a clear distinction between the LESCs and our control CECs. The genes that were mostly affected were involved in ion-, nucleotide- or protein binding, protein secretion as well as receptor or enzyme activities. Table 3 shows the top 20 up- or down-regulated genes within these gene groups.

**Table 3:** Top 20 up- and down-regulated custom selected genes in LESC

**Symbol**	**Entrez gene name**	**Fold change**	**Activation z-score**	**p-value of overlap**	**Molecule type**
**Fold change up-regulated**					
*FN1*	Fibronectin 1	74.934	2.979	8.37E-05	Enzyme
*CCNA1*	Cyclin A1	27.199		3.42E-02	Other
*IL1B*	Interleukin 1, beta	24.948	4.924	8.09E-15	Cytokine
*INHBA*	Inhibin, beta A	21.815	1.352	2.27E-04	Growth factor
*SERPINE1*	Serpin peptidase inhibitor, clade E (nexin, plasminogen activator inhibitor type 1), member 1	18.854	−0.927	1.40E-02	Other
*GDF15*	Growth differentiation factor 15	16.924	1.999	2.63E-02	Growth factor
*PTHLH*	Parathyroid hormone-like hormone	16.2	1.972	8.72E-03	Other
*OSMR*	Oncostatin M receptor	15.366	1.982	1.83E-02	Transmembrane receptor
*CXCL10*	Chemokine (C-X-C motif) ligand 10	15.171	0.911	2.31E-02	Cytokine
*MMP9*	Matrix metallopeptidase 9 (gelatinase B, 92 kDa type IV collagenase)	14.243	0.689	1.72E-02	Peptidase
*IL1R1*	interleukin 1 receptor, type I	13.972	2.603	5.76E-03	Transmembrane receptor
*MMP1*	Matrix metallopeptidase 1 (interstitial collagenase)	13.875	1.188	4.01E-03	Peptidase
*ICAM1*	Intercellular adhesion molecule 1	13.681	2.961	1.36E-03	Transmembrane receptor
*ITGA5*	Integrin, alpha 5 (fibronectin receptor, alpha polypeptide)	13.455	2.411	1.46E-02	Other
*SH3KBP1*	SH3-domain kinase binding protein 1	12.752		4.98E-02	Other
*AKT3*	V-akt murine thymoma viral oncogene homolog 3 (protein kinase B, gamma)	11.843		4.76E-02	Kinase
*LOXL2*	Lysyl oxidase-like 2	11.734	1.992	1.88E-03	Enzyme
*CEACAM5*	Carcinoembryonic antigen-related cell adhesion molecule 5	10.588		1.23E-02	Other
*SLPI*	Secretory leukocyte peptidase inhibitor	8.53	−2.433	1.06E-02	Other
*PDZK1IP1*	PDZK1 interacting protein 1	8.485		1.23E-02	Other
**Fold change down-regulated**					
*CRTAC1 -*	Cartilage acidic protein 1	*−72.277*			
*LPA*	Lipoprotein, Lp(a)	−11,623		4.98E-02	Other
*ETV1*	Ets variant gene 1	−7,444	1.969	1.83E-02	Transcription regulator
*EDNRB*	Endothelin receptor type B	−7,25		3.38E-02	G-protein coupled receptor
*BMP7*	Bone morphogenetic protein 7	−6,436	0.733	1.17E-04	Growth factor
*NREP*	Neuronal regeneration related protein	−5,823	−0.248	3.81E-03	Other
*CFTR*	Cystic fibrosis transmembrane conductance regulator (ATP-binding cassette sub-family C, member 7)	−5,766	−1.993	1.49E-01	Ion channel
*DCN*	Decorin	−5,066	0.172	6.04E-07	Other
*RORA*	RAR-related orphan receptor alpha	−4,781	−0.439	2.61E-03	Ligand-dependent nuclear receptor
*LEF1*	Lymphoid enhancer binding factor 1	−4,441	−0.306	2.01E-02	Transcription regulator
*BDKRB1*	Bradykinin receptor B1	−4,1	−2.000	6.89E-04	G-protein coupled receptor
*GJA1*	Gap junction protein, alpha 1	−3,94	−1.480	5.54E-04	Transporter
*FAM3B*	Family with sequence similarity 3, member B	−3,9		3.20E-08	Cytokine
*P2RX7*	Purinergic receptor P2X, ligand-gated ion channel, 7	−3,885		1.15E-02	Ion channel
*KAT2B*	K(lysine) acetyltransferase 2B	−3,829	1.963	8.27E-02	Transcription regulator
*ODC1*	Ornithine decarboxylase, structural 1	−3,63		4.98E-02	Enzyme
*EPHX2*	Epoxide hydrolase 2, cytoplasm	−3,469		3.42E-02	Enzyme
*MAT1A*	Methionine adenosyltransferase I, alpha	−3,386	−0.215	1.82E-02	Enzyme
*CTSL2*	Cathepsin L2	−3,385		4.98E-02	Peptidase
*DUSP1*	Dual specificity phosphatase 1	−3,358	−1.881	6.12E-02	Phosphatase
*NOV*	Nephroblastoma overexpressed	−3,149	0.555	1.94E-02	Growth factor

The top 20 up and down regulated genes in LESCs as determined by the IPA software.

### Customized gene networks – upstream regulators

#### Stemness and proliferation

**Figure 2:**
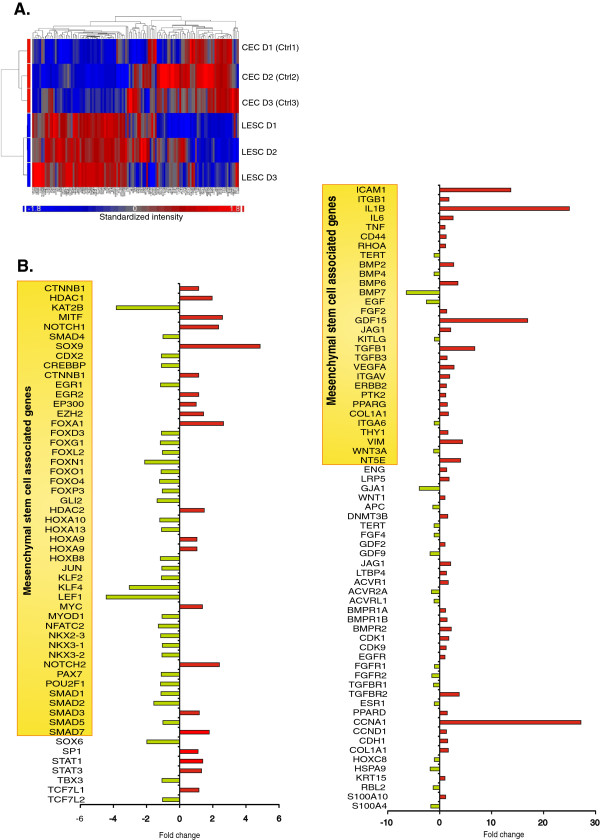
**Upstream regulators as determinants for stemness.** Selected upstream regulator genes which are involved in the maintenance of stemness, cell cycle and multipotency-related transcription factors **(A)**, and growth factors, cytokines and corresponding receptors **(B)**. The gene characteristics of MSCs are highlighted as well.

As seen in Figure 2, out of the 257 upstream regulators, 122 were related to stemness and, in particular, mesenchymal stem cells (MSCs). The expression pattern also demonstrated a clear difference between the LESCs and the CECs (Figure 2A). These genes coded for transcription factors, surface molecules, cytokines and growth factors, all playing a key role in the maintenance of multipotency, proliferation capacity of hematopoietic and/or MSCs (Figure 2B). Up- and down- regulation was found in 66 and 56 genes, respectively, and within the custom selected gene cluster, the 10 highest upstream regulators were: *CCNA1* (27.199 fold), *IL1B* (24.948*), GDF15* (16.924), *ICAM1* (13.681), *TGFB* (16.745) *SOX9* (4.859 fold) *VIM* (4.368), *NT5E* (4.009) *TGFBR2* (3.772) and *BMP6* (3.494), while the 10 most down-regulated were: *BMP7* (−6.436 fold), *LEF1* (−4.441), *GJA1* (−3.94), *KAT2B* (−3.829), *KLF4* (−3.041), *EGF* (−2.563) *FOXN1* (−2.11), *SOX6* (−1.984), *GDF9* (−1.865) and *HSPA9* (−1.838). The expression of these selected genes strengthen our previous findings that the *ex vivo* cultured LESCs show great similarity to MSCs regarding their surface marker profile and the extracellular matrix (ECM) production ability [13]. The present comparison is rather focused on the differences between LESCs and differentiated CECs in their transcriptional factor patterns, making the LESCs more progenitor-like, yet with a limited multipotency potential as compared to other stem cells, including bone marrow-derived MSCs (bmMSCs). As expected, our data show that LESCs have a higher proliferation potential and stemness-related gene expression than differentiated CECs. The SRY related HMG-box family members *SOX9* and *SOX6*, both involved in chondrogenesis and proliferation, were down-regulated in the LESCs. Flow cytometric surface protein level analysis found a significantly higher number of positive cells for ICAM1 in CECs (56.19 ± 12.46%) than in LESCs (4.37 ± 7.63%) (p = 0.0001) (Additional file 1: Figure S1). No difference could be found in the well-known MSC surface markers, such as CD90 (8.75 ± 19.56% in LESCs and 1.77 ± 3.54% in CECs, respectively (p = 0.4748)) and CD73 (89.86 ± 6.15% in LECSs and 76.93 ± 17.43% in CECs, respectively, (p = 0.2374); data shown are Mean ± SD), while more cells expressed the stem cell factor receptor CD117/c-kit in the LESCs (19.87 ± 24.92%) compared to CECs (0 ± 0%) at a protein level, however, this difference was not statistically significant (p = 0.1491) due to a high inter-donor variance (Additional file 1: Figure S1).

#### *Differentiation*

**Figure 3:**
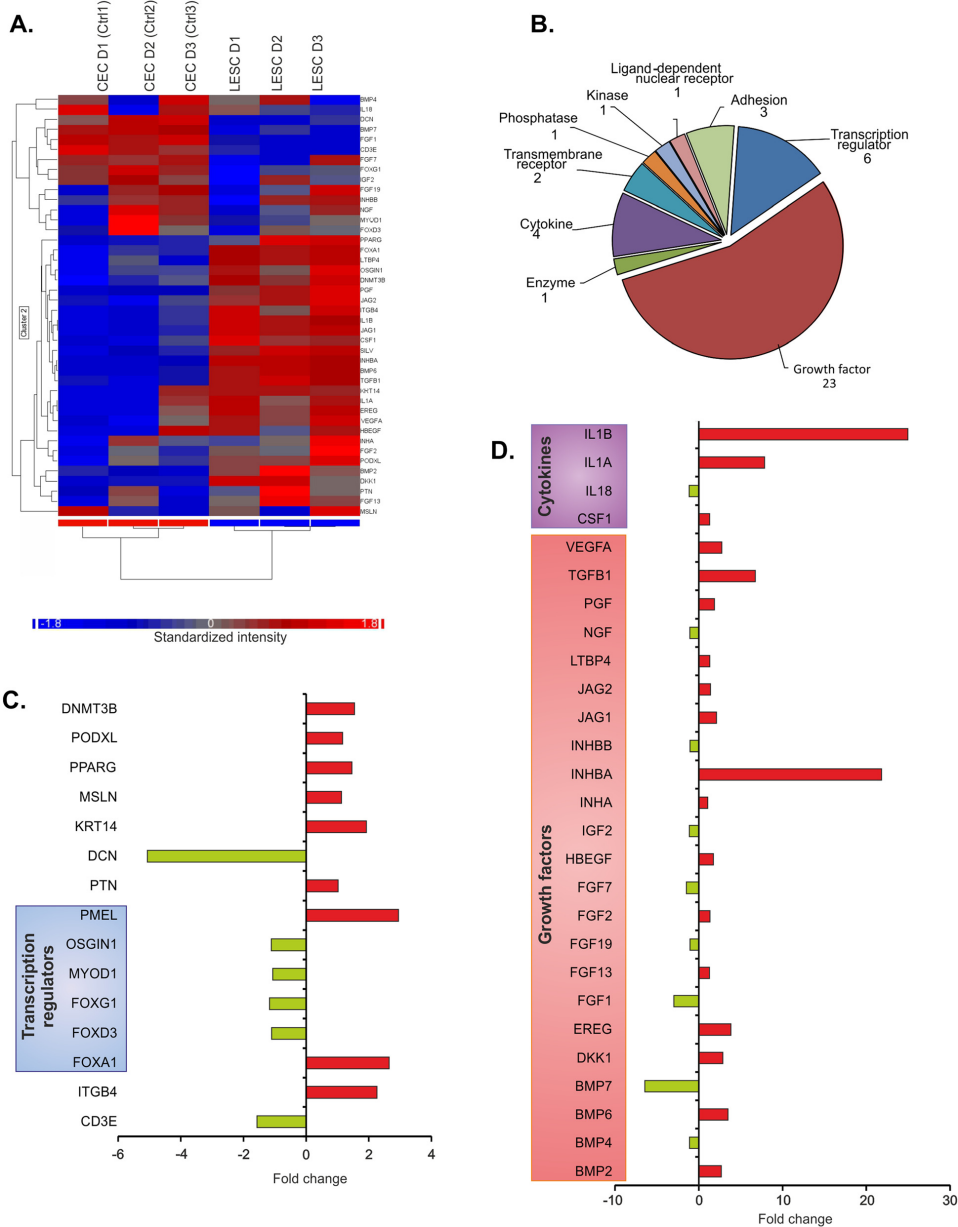
**Expression of terminal differentiation related genes.** Expression of transcription factors, transmembrane receptors, enzymes and adhesion molecules **(A)**. Subgroups of cytokines- and growth factor coding genes involved in epithelial differentiation of stem cells **(B)**. Distribution of the selected 42 upstream regulators by molecule type, such as transcriptional regulators **(C)** and growth factors and cytokines **(D)**.

Our previous phenotype analysis of LESCs showed the heterogeneity of this population [13], so we analyzed 42 genes related to terminal and epithelial differentiation. The heatmap of these transcripts with the clustering of the expressed genes show a clear segregation of the LESCs from the differentiated CECs (Figure 3A). Among them, growth factors, cytokines, adhesion molecules, transcription regulators and enzymes can be found (Figure 3B). Transcriptional regulators such as *FOXG1* (−1.165), *FOXD3* (−1.1), *MYOD1* and *OSGIN1* (−1.109) were all down-regulated in contrast to the *FOXA1* and *PMEL* up-regulation (Figure 3C). The pericellular matrix proteoglycan decorin coding gene *DCN* (−5.066) was found to be down-regulated in LESCs. Within the collection of cytokines and growth factors which play a role in epithelial differentiation, *BMP7* (−6.436 fold), *FGF1* (−2.96), *FGF7* (−1.473), *IL18* (−1.152) and *IGF2* (−1.126) were down-, while *IL1B* (24.948), *INHBA* (21.815), *IL1A* (7.853), *TGFB1* (6.745), *EREG* (3.836), *BMP6* (3.494) and *DKK1* (2.88) were up-regulated (Figure 3D). At a protein level, CD146/MCAM, a key player in MSCs differentiation, was found not to be expressed on the surface of CECs (0 ± 0%) compared to LESCs (82.40 ± 14.60%, p = 0.0002) (Additional file 1: Figure S1). Presence of CK14 on LESCs has been described by our group previously [13].

#### *Cell adhesion*

**Figure 4:**
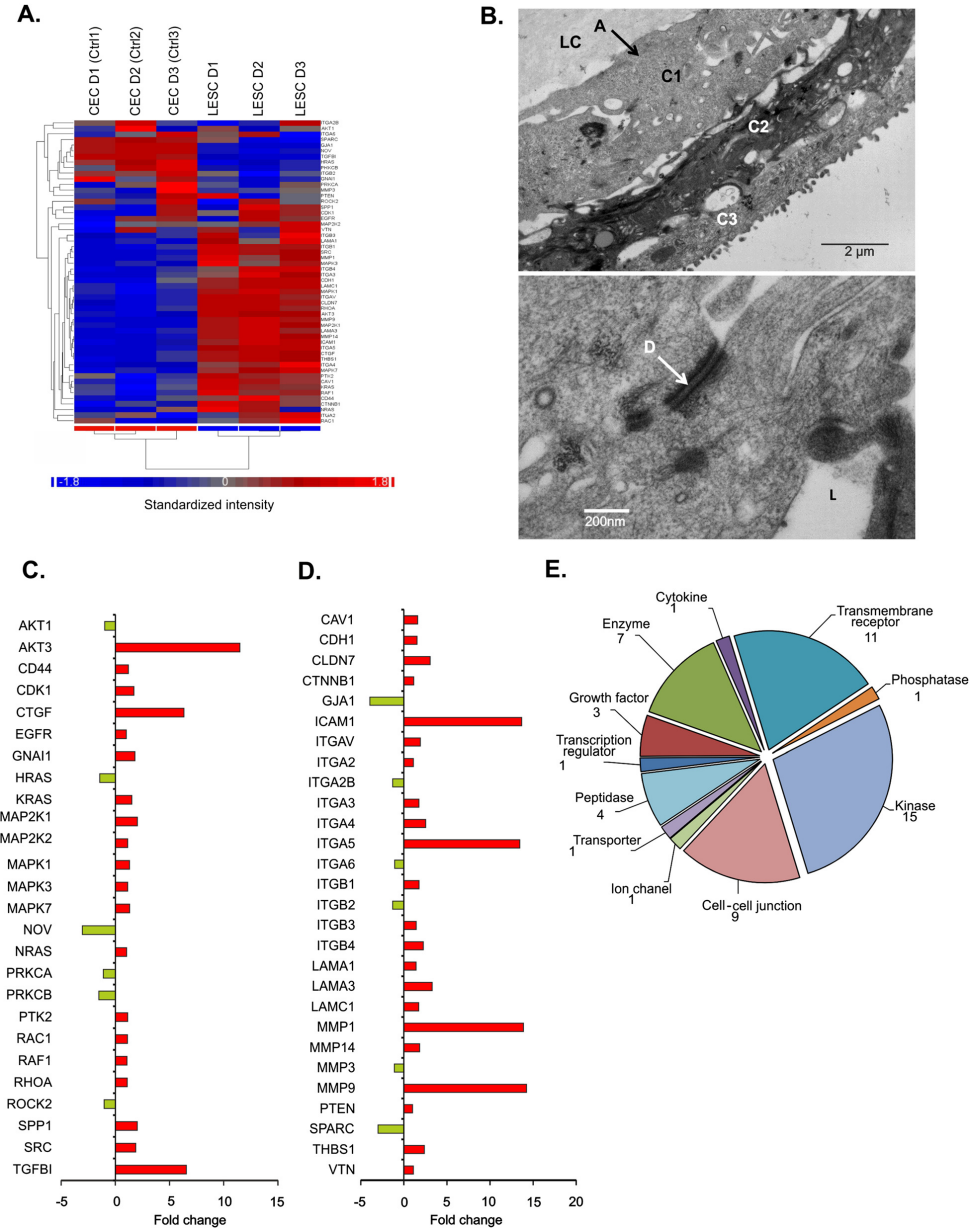
**Differential changes in selected genes related to cell-cell junction, cell-to-cell and cell-to-extracellular matrix (ECM) adhesion.** Collection of significantly different expressions of transcription factors, kinases and transmembrane receptors related to cell-cell connection and adhesion **(A)**. CAMs, integrins and ECM receptors determine the tissue origin of LESCs **(C and D)**. Molecule types of the selected 54 upstream regulator in the two groups of cells **(E)**. TEM pictures about LESCs on lens capsule shows the cell-cell junctions between the cells **(B)** (LC = Lens capsule, A = Attachment between LC and cells; C1, ^,^, C2 and C3 - three cell-layer, L = translucent space, **D** = Desmosomes.

To further distinct the multipotent LESCs within the heterogeneous population of epithelial cells, surface markers including ECM-cell, cell-cell adhesion and cell migration proteins were used as putative markers. The upstream regulators of 54 genes coding for molecules involved in cell adhesion were analyzed. (Figure 4A, E). The first subgroup contained highly expressed transcriptional factors and transmembrane receptors in the LESCs (Figure 4C): *TGFBI* (6.745), *AKT3* (11.843), *CTGF* (6.513), *MAP2K* (12.088), *SPP1* (2.077) and *SRC* (1.931). Six genes including *AKT1* (−1.026), *NOV* (−3.149), *ROCK2* (−1.076), *PRKCA* (−1.154), *HRAS* (−1.5) and *PRKCB* (−1.583) were down-regulated. The next subgroup (Figure 4D), included integrins, cell adhesion molecules (CAMs), proteolytic enzymes and matrix metalloproteases (MMPs) – all involved in the ECM breakdown and tissue healing and remodeling; the most up-regulated genes in this cluster were *MMP1* (13.875), *MMP14* (1.836) and *MMP9* (14.243), while *MMP3* was down-regulated (−1.105). The laminins, which are important proteins in the basal membrane and their coding genes such as *LAMA1* (1.428), *LAMA3* (3.289) and *LAMC1* (1.724) were all up-regulated in the LESCs. CAMs and tight junctions which are very important in cell-cell adhesion and tissue organization, such as *ICAM-1* (13.681), *CAV1* (1.608) and *CLDN7* (3.056) were up-, while *GJA1* (−3.94) was down- regulated. In particular*, CDH1* (1.536), important in desmosomal junction formation and stratified epithelium transformation was up-regulated, and the desmosome formation between the LESCs grown on lens capsule could be demonstrated using transmission electron microscopy (Figure 4B). Altogether, the expression of 11 integrin-coding genes was different between the LESCs and the differentiated CECs - 8 out of them were up-, while 3 were down- regulated. Surface protein level analysis found no difference between LESCs and CECs in the expression of CD29/Integrin β1 (97.01 ± 1.87% and 78.28 ± 15.84%, respectively), and CD44/HCAM (16.55 ± 23.21% and 19.83 ± 21.55%, respectively) expression. The percentage of CD47 positive cells, which plays a role in cell viability and immunoregulation, was significantly higher in LESCs (98.98 ± 0.57%) compared to CECs (25.9 ± 27.44%) (p = 0.0039), showing higher viability and inhibition of phagocytosis in the LESCs (Additional file 1: Figure S1).

#### Cytokines and growth factors

**Figure 5:**
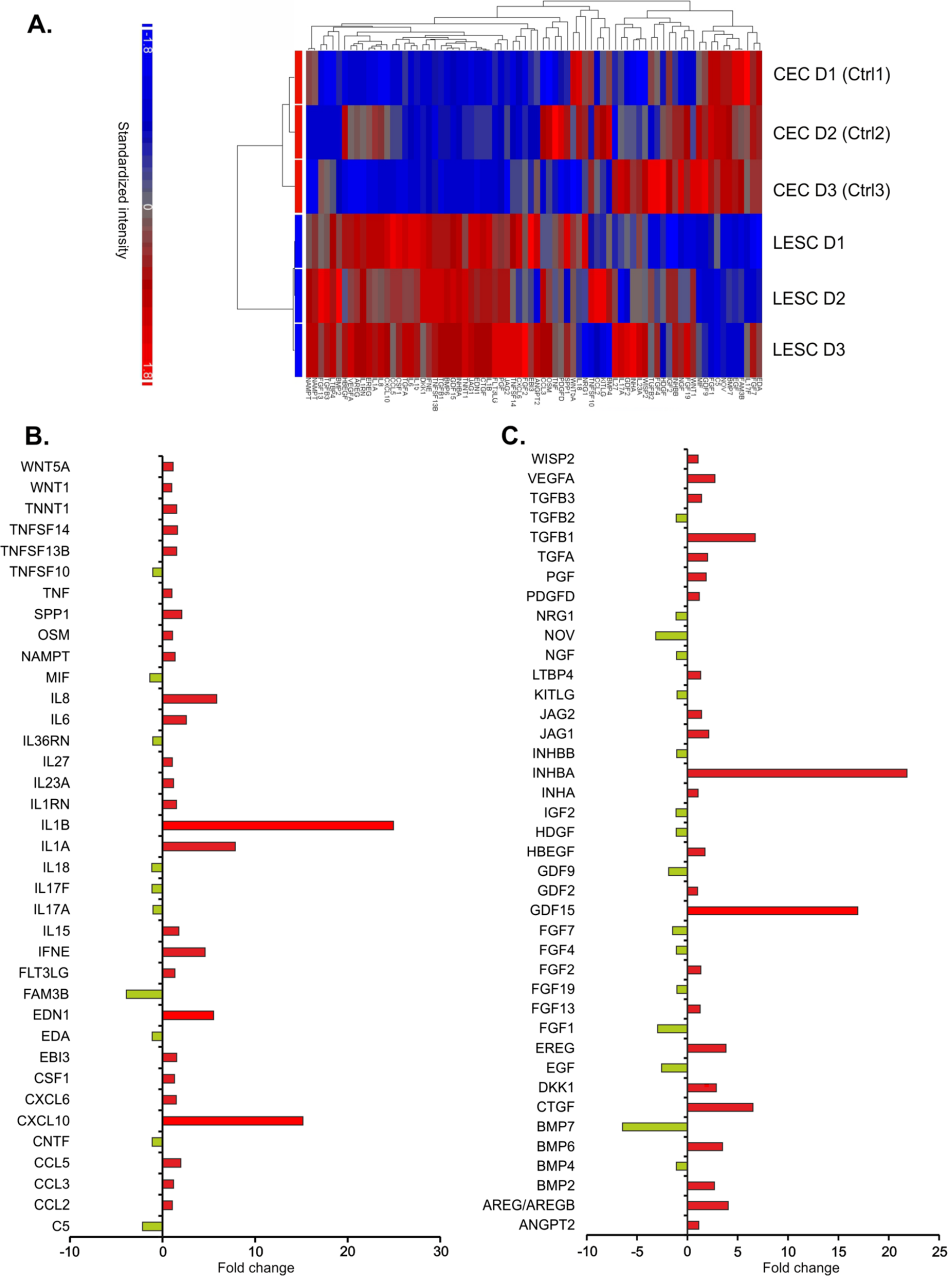
**Differences in the expression of the cytokines and growth factors coding genes.** Heatmap of the transcripts of cytokines- and growth factors- coded genes in LESCs and CECs **(A)**. Selection of significantly and differentially expressed genes of cytokines **(B)**; and differentiation and growth factors **(C)**. In comparison to CECs, the LESCs expressed 37 cytokine and 40 growth factor coding genes in a significantly different manner.

Cytokines and growth factors have an important function in cell-cell communication and can affect cell function, differentiation and immunogenicity (Figure 5A). *IL1B* was the most up-regulated gene (24.948 fold), followed by *CXCL10* (15.171), *IL1A* (7.853), *IL8* (5.849), *EDN1* (5.504), *IFNE* (4.601), *IL6* (2.57), *SPP1* (2.077) and *CCL5* (1.973). Although most of the up-regulated genes were related to pro-inflammatory cytokines, some members with similar pro-inflammatory properties, but from other cytokine families, were down-regulated: *IL17* (−1.129), the IL-1 superfamily members *IL18* (−1.152) and *IL36RN* (−1.059). Human *EDA* (−1.113) which belongs to the TNF family was within the most down-regulated genes, while the top down-regulated gene was *FAM3B* (−3.900) (Figure 5B). Next, we filtered out the entire dataset for growth factors, all being important for maintaining multipotency and differentiation of progenitor or stem cells (Figure 5C). The most up-regulated genes were members of the TGF beta (TGFβ) superfamily: *INHBA* (21.815 fold), *GDF15* (16.924), *TGFB1* (6.745) and *BMP6* (3.494). Epiregulin and amphiregulin, members of the epidermal growth factor (EGF) family, were the top up-regulated genes: *EREG* (3.836) and *AREG* (4.047), as well as connective tissue growth factor *CTGF* (6.513). The down-regulated genes included other TGFβ superfamily members: *BMP7* (−6.436) and *GDF9* (−1.865). Acidic fibroblast growth factor, *FGF1* (−2.96) and *FGF7* (−1.473) were also down-regulated, as well as NOV-like CTGF- member of the CCN protein family: nephroblastoma overexpressed/*NOV* (−3.149). Similarly, EGF gene expression responsible for regulation of cell division and proliferation was down-regulated −2.563 fold.

#### *Angiogenesis*

**Figure 6:**
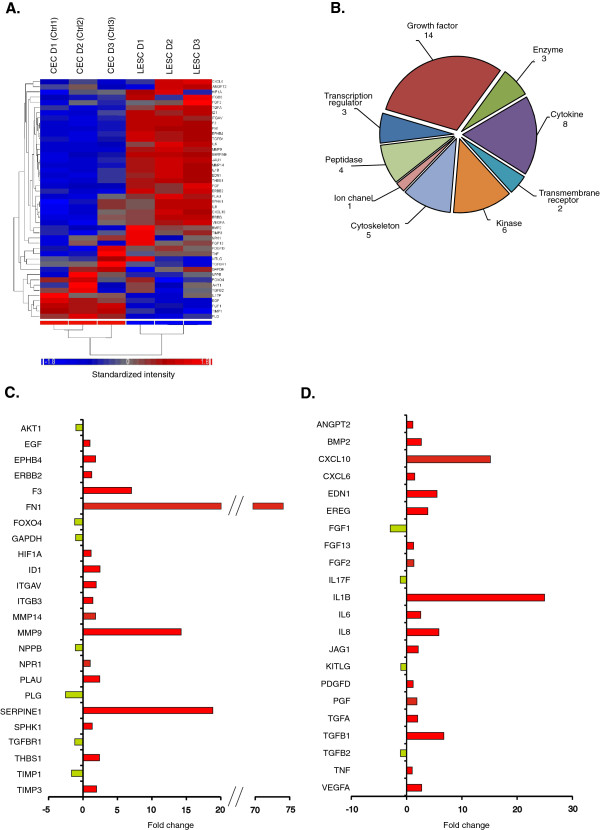
**Significantly expressed angiogenesis-related genes in LESCs.** Angiogenesis is a complex process mediated by MMPs, proteolytic enzymes and ECM proteins **(A)**. Cytokines and growth factors are important players of angiogenesis with endothelial cell activation and EPC/stem cell differentiation **(B)**. Most of the angiogenic molecules belong to these molecule types **(C and D)**.

48 molecules were detected in the dataset which may have a role in pathological angiogenesis in the cornea (Figure 6A). This set contained transcription factors, enzymes and cytokines including angiogenic growth factors as well (Figure 6B). The fibronectin gene (*FN1),* which is important in new vessel sprout formation, had the highest up-regulation (74.934), followed by *SERPINE1* (18.854) and *MMP9* (14.243). The coagulation factor III (thromboplastin) gene *F3* (7.054) was also highly expressed in the LESCs. The most down-regulated genes were *PLG* (−2.521), *TIMP1* (−1.658), *FOXO4* (−1.213), *TGFBR1* (−1.179) (Figure 6C). Certain cytokines and growth factors which are also important in angiogenesis (Figure 6D) were up-regulated in the LESCs: *ILB1* (24.948), C-X-C motif chemokine 10, *CXCL10* (15.171), *TGFB1* (6.745) and *VEGFA* (2.742). In addition, IL-6 and IL-8, two very potent angiogenic cytokines, were up-regulated in these cells: *IL-6* (2.57) and *IL-8* (5.849), similar to *EDN1* (5.504), *EREG* (3.836) and *BMP2* (2.686) up-regulation within this cluster. Only four of the angiogenic cytokines were down-regulated in the LESCs: acidic FGF - *FGF1* (−2.96), *IL17F* (−1.129), *TGFB2* (−1.106) and *KITLG* (−1.015).

## Discussion

Absence or removal of the LESC layer in animals can cause defective corneal epithelialization, indicating the essential importance of these cells in corneal surface biology and regeneration [15,16]. In humans, besides injuries and diseases, dysfunction or LESC loss can lead to LESCD; other causes, such as genetic diseases can cause abnormal development of the anterior segment and the limbus, while Steven Johnson syndrome, chronic limbitis or ocular pemphigoid are inflammatory processes which can lead to LESCD, similar to cytotherapy, radiation or surgery in the limbal region [9,17]. Altogether, a plethora of causes can lead to decreased transparency of the cornea, inflammation and corneal neovascularization (key features of LESCD), resulting in a serious and painful disease with subsequent loss of vision [18]. The inflammatory processes and the angiogenesis very likely change the environment, so that the small niche of stem cells becomes non-functional [6,10,19]. Therefore, the treatment of LESCD with *ex vivo* cultured and functional LESCs is becoming widely accepted today [20]. Many other types of cells, including embryonic stem cells, bone-marrow and Wharton jelly-derived stem cells have been attempted for LESCD treatment in animal models with relatively good outcomes [17,21]. Most cell-based therapies in the clinical practice, however, use limbal epithelial cells cultured on 3 T3 mouse feeder fibroblast supplemented with fetal calf serum [22]. The risk of murine (animal) viral transmission during such procedures is not yet known [23,24]. Although the limbal epithelial cells cultivated on mouse feeder cells can replace the wounded epithelial cells, the mechanism how they make the local tissue more suitable for its own stem cells to recover their stemness and differentiation potential has been unknown [17]. This seemingly “gold standard” cell therapy method would not be able to compete with human animal material-free product that would be ideal for clinical use. Furthermore, the overall success rate of the above therapies has been reported to be 76% [24], although, the right quantity of cells needed for recovery has not yet been reported. In stem cells based therapies, the purity of the product (the percentage of stem cells within the cell culture) is crucial for the outcome [24-26]. LESCs lose their multipotency during epithelial expansion and differentiation [27], therefore, it is important to distinguish between LESCs, TAMs and CECs within the cell culture used for therapy. In our cell cultures, the SRY related HMG-box family member SOX9 was up-regulated, while SOX6 expression was down-regulated, indicating no chondrogenic differentiation but high proliferative capacity of the LESCs. Furthermore, S100A4 and A9 proteins have been found to be potent markers of limbal epithelial crypt cells [28] - in our LESCs,- the *S100A4* was down-regulated indicating they are not crypt cells. Others have reported that *CXCL12*, *COL2A*, *ISL1*, *FOXA2*, *NCAM1*, *ACAN*, *GJB1* and *MSX1* can be used as putative markers to identify LESCs [29]. We could not confirm a difference in these genes between the LESCs and the differentiated CECs, with the exception of *FOXA1* which was up-regulated and *GJA1* down-regulated (also known as negative marker for LESCs [30]). Similarly, Wnt2, Wnt6, Wnt11 and Wnt16b have been reported to be typically expressed in the limbal region and to be important for the LESCs proliferation [31]. We could confirm that *WNT1* and *WNT5A* expression was up-, while *WNT3A* was down- regulated in our LESCs, along the wider lines of the results mentioned above. Surface protein level analysis found higher positivity for CD146/MCAM, CD47 and CD117/c-kit in LESCs compared to CECs, showing a pattern typical for stem cells and higher multipotency in the earlier cell type (Additional file 1: Figure S1). This phenotype analysis further proved simply using classical MSC markers, such as CD90/Thy-1 and CD73, it is not possible to differentiate between the two cell types.

LESCs play a key role in limbal tissue healing and remodeling, a process which usually starts with ECM breakdown [32,33]. The latter is mediated by MMPs, which were up-regulated in the LESCs and their pattern implicates a preferred degradation of collagens to rebuild the ECM [32]. Laminins and vitronectin are typically found in the limbal basal membrane [34,35] and their genes were up-regulated in our LESCs.

For attachment to new ECM proteins, integrins and CAMs are also essential, the expression of which is typical for the tissue of origin. Indeed, the integrin expression is able to define the cell phenotype and seems to be useful in classifying MSCs from various tissues besides the well-known MSC markers we have reported before [13]. The results of our gene array data analysis strengthen the fact that LESCs cultured in medium containing human serum as the only growth supplement can keep their integrin and CAM pattern that relates them to their limbal tissue phenotype. Surface protein level analysis found same expression levels of CD29/Integrinβ1 and CD44/HCAM in the two cell types, while CD54/ICAM1 positivity was higher in the CECs (Additional file 1: Figure S1).

Wound healing can often lead into angiogenesis, which can have a very important and controllable pathological role in the limbus [33,36-38]. Fibronectin is an important ECM protein in expanding cells as well as angiogenesis, mediating sprouting, *de novo* vessel formation and endothelial progenitor/stem cells differentiation into endothelial cells [39-41]. The two highest up-regulated gene products found in our LESCs seem to have an opposite effect on the angiogenesis pathway: IL-1β can induce-, while CXCL10 can inhibit the formation of new vessels [42-46]. Interestingly, human limbal epithelial progenitor cells have been found to express CXCL10 [47] while its absence could decrease the level of IL-6 in mice corneas [48]. The expression of *TGFB1* is very important in wound healing and in inducing *VEGF* expression, which was also up-regulated in the LESCs, capable of provoking angiogenesis in the damaged tissue [49,50]. Endothelin-1 has many direct and indirect angiogenic effects upon the endothelial cells and fibroblasts - it provokes the release of the pro-angiogenic compounds like VEGF from endothelial cells and stimulates the fibroblasts to produce pro-angiogenic proteases [51,52]. Altogether, our results indicate that both pro- and anti-angiogenic genes are expressed at the same time or in a balanced way in LESCs, maintaining an avascular state in the normal cornea. Loss of this control can be initiated by either a decreased production of anti-angiogenic molecules or increased production of pro-angiogenic and inflammatory factors. Although transplantation of LESCs has been known to suppress corneal inflammation and angiogenesis, the molecular mechanism how LESCs participate in the processes has not yet been fully understood [9,17,36,53,54]. Limbal niche cells have been found to have a differentiating ability towards angiogenic progenitors and inhibition of endothelial differentiation of LESCs [53].

IL-6 and IL-8 can be secreted by many cell types during inflammation or differentiation. These cytokines play a role in inflammation, angiogenesis and MSC differentiation-related processes [55]. Their gene expressions were up-regulated in LESCs: *IL-6* (2.570) and *IL-8* (5.849). Using the IPA analysis, the IL-6 signaling pathways were further confirmed of being present in our LESCs compared to CECs, together with some other well-known pathways described below (Additional file 2: Figure S2A). The first such pathway or network affected is the IL-1β and TNFα mediated release of IL-6 from activated cells. This signaling is further mediated by NFκB and JNK (JUN, C-Fos) transcriptional factors and can lead to IL-6 and IL-8 release in parallel to collagen type I production (COL1A1), which is the major component of connective tissue. The second network affected is the autocrine or IL-6-mediated-IL-6-secretion through RAF1, MAP2K and ERK1/2. This process needs to be initiated by the IL-6 receptor (IL6R), however, the JAK-STAT pathway (STAT3) can also induce release of angiogenic factors such as VEGF and activation of SOX3. As shown before in our dataset, *IL1B* was highly up-regulated with a 24.948 fold change hand-in-hand with its receptor *IL1R1* (13.972) and *IL1A* (7.853). Although a subunit of the receptor for IL-6 coding gene was down-regulated - *IL6R* (−2.640), a member of the type I cytokine receptor family - oncostatin M receptor (*OSMR*), was found to be highly up-regulated (15.366) in the LESCs. This receptor can form heterodimers with gp130, which is a signal transducer for IL6R. It can also provide an intracellular signal through Janus kinases after ligand binding. In addition, many other ligands can be associated with gp130 (and the IL6 receptor as well) such as IL-11, ciliary neurotrophic factor (CNTF), leukemia inhibitory factor (LIF) and cardiotrophin 1 (CT-1). Activation of RAS and MAPK signaling can then be connected to the IL-1β mediated pathway. In our dataset, *SOCS3* was up-regulated (1.397), while *SOCS1* was down-regulated (−1.120). Four MAPKs were slightly up-regulated in the LESCs: *MAP2K1* (2.088), *MAPK1* (1.339), *MAPK14* (1.011) and *MAPK3* (1.163), while the members of the NFκB pathway were down-regulated: *NFKB1* (−1.178) and *NFKBIA* (−1.193). CXCL10 with high amount of IL-6 has been shown to induce migration of trophoblasts through activation of the CXCR3 receptor [56]. Interestingly, *CXCL10* was among the highest up-regulated genes (15.171) in the LESCs compared to CECs (Additional file 2: Figure S2B).

The pathways in which IL-8 participates are in general more complex than for other cytokines. IL-8 can be produced by any cell possessing toll-like receptors during inflammation, and it is one of the most known chemotactic factors for neutrophils and activator of immune cells [57]. (Additional file 3: Figure S3A). In addition, IL-8 has been described as potent pro-angiogenic cytokine especially in the eye [58,59], although the molecular background of such angiogenic processes has not been well described. IL-8 can bind to G protein-coupled serpentine receptors such as CXCR1 and CXCR2 and beside immunological activation, it can induce rearrangement of cytoskeletal proteins, increase the expression of VCAM and ICAM1, and the migration as well as vessel formation of endothelial cells and stem cell-like endothelial progenitor cells, in parallel with increase in vascular permeability (Additional file 3: Figure S3B).

Our gene expression data which indicate that IL-6 and IL-8 participate in most of the networks or selected pathways analyzed correlate well with the measurements of their secreted levels in the supernatants of the cultured LESCs. The level of these cytokines was continuously high in the culture supernatants at days 9 (5885.24 ± 685.6 pg/mL) and 13 (6147.14 ± 530.21 pg/mL) with no significant difference at both time points (p = 0.14) (Additional file 4: Figure S4). For comparison, the physiologic level of IL-6 in the tear fluid of human subjects is very low (2.4 pg/mL) (data from our group under publication). IL-6 can participate in many stem cell-related processes and has been found to be important in maintaining the needed niche for LESCs and LESC-epithelial interaction [60]. In bone marrow-derived stem cells, IL-6 is needed for immunosuppression, which effect of the LESCs has been described with different possible mechanisms [11].

Overall, our gene selection and networks are somewhat different from the well-known canonical pathways described so far because they were generated *de novo* and were based on our data and the already published networks from literature. It remains to be further investigated and confirmed whether these pathways are reflected in the same manner at protein level both *ex vivo* and *in situ*, giving a possibility of finding a specific phenotype and genotype profile for LESCs. These can clearly be beneficial in treating ocular surface diseases and discovering innovative therapies aided by the gene array technology.

## Conclusion

The human eye as an organ possesses great potential for regeneration and cell therapy, in particular, its corneal surface which contains LESCs. Identifying molecular markers and upstream regulators in the LESCs using genome-wide microarray transcriptional profiling, as well as verification of those at protein expression level can provide a better identification and more specific understanding of the signaling molecules associated with these cells, therefore, better application ocular surface disease treatment. Overall, we found that the human LESCs play a crucial role in cellular movement and adhesion, epithelial differentiation and tissue repair, as well as angiogenesis and extracellular matrix integrity.

## Abbreviations

ACAN: Aggrecan/cartilage-specific proteoglycan core protein (CSPCP)/chondroitin sulfate proteoglycan 1; AKT1: RAC-alpha serine/threonine-protein kinase; AKT3: RAC-gamma serine/threonine-protein kinase; AREG: Amphiregulin; BMP2: Bone morphogenetic protein 2; BMP6: Bone morphogenetic protein 6; BMP7: Bone morphogenetic protein 7; CAMs: Cell adhesion molecules; CAV1: Caveolin-1; CCL5: Chemokine (C-C motif) ligand 5/RANTES; CCNA1: Cyclin-A1; CDH1: Cadherin/ CAM 120/80 or epithelial cadherin; CECs: Corneal epithelial cells; CLDN7: Claudin-7; CNTF: Ciliary neurotrophic factor; COL2A: Collagen, type II, alpha; CT-1: Cardiotrophin 1; CTGF: Connective tissue growth factor; CXCL10: C-X-C motif chemokine 10/interferon gamma-induced protein 10; CXCL12: Chemokine (C-X-C motif) ligand 12/ stromal cell-derived factor-1; CXCR1: Interleukin 8 receptor, alpha; CXCR3: Chemokine receptor CXCR3; DCN: Decorin; DKK1: Dickkopf-related protein 1; ECM: Extracellular matrix; EDA: Ectodysplasin-A; EDN1: Endothelin 1/ preproendothelin-1; EGF: Epidermal growth facto; ELISA: Enzyme-linked immuno sorbent assay; EREG: Epiregulin; ERK1/2: Extracellular signal-regulated kinases; F3: Platelet tissue factor, factor III, thromboplastin; FAM3B: Protein FAM3B; FC: Fold change; FGF1: Heparin-binding growth factor 1; FGF7: Keratinocyte growth factor; FOXA1: Forkhead box protein A1/hepatocyte nuclear factor 3-alpha; FOXA2: Forkhead box protein A2/hepatocyte nuclear factor 3-beta; FOXD3: Forkhead box D3; FOXG1: Forkhead box protein G1; FOXN1: Forkhead box protein N1; FOXO4: Forkhead box protein O4; GDF15: Growth differentiation factor 15; GDF9: Growth differentiation factor 9; GJA1: Gap junction alpha-1 protein/connexin 43; GJB1: Gap junction beta-1 protein/connexin 32; gp130: Glycoprotein 130; HCAM: Homing-associated cell adhesion molecule; HRAS: Transforming protein p21; HSPA9: Stress-70 protein, mitochondrial; ICAM1: Intercellular Adhesion Molecule (CD54); IFNE: Interferon epsilon; IGF2: Insulin-like growth factor 2; IL-11: Interleukin 11/adipogenesis inhibitory factor; IL17: Interleukin 17; IL17F: Interleukin 17 F; IL18: Interleukin-18/interferon-gamma inducing factor; IL1A: Interleukin-1 alpha; IL1B: Interleukin 1 beta; IL1R1: Interleukin 1 receptor, type I; IL36RN: Interleukin 36 receptor antagonist; IL-6: Interleukin 6; IL6R: Interleukin 6 receptor (CD126); IL-8: Interleukin 8; INHBA: Inhibin, beta A; IPA: Ingenuity Pathway Analysis; IPKB: Ingenuity Pathways Knowledge Base; ISL1: Insulin gene enhancer protein; JAK: Janus kinase; KAT2B: P300/CBP-associated factor (PCAF/K(lysine) acetyltransferase 2B; KITLG: Stem Cell Factor; KLF4: Kruppel-like factor 4; LAMA1: Laminin subunit alpha-1; LAMA3: Laminin subunit alpha-3; LAMC1: Laminin subunit gamma-1; LEF1: Lymphoid enhancer-binding factor 1; LESCD: LESC deficiency; LESCs: Limbal epithelial stem cells; LIF: Leukemia inhibitory factor; MAP2K: Mitogen-activated protein kinase kinase; MAP2K1: Dual specificity mitogen-activated protein kinase kinase 1; MAPK: Mitogen-activated protein kinases; MAPK1: Mitogen-activated protein kinase 1; MAPK14: Mitogen-activated protein kinase 14/p38-α; MAPK3: Mitogen-activated protein kinase 3; MCAM: Melanoma cell adhesion molecule; MMP1: Matrix metalloproteinase-1/interstitial collagenase; MMP14: Matrix metalloproteinase-14; MMP3: Stromelysin-1/matrix metalloproteinase-3; MMP9: Matrix metallopeptidase 9; MMPs: Matrix metalloproteases; MSCs: Mesenchymal stem cells; MSX1: Msh homeobox 1; MYOD1: MyoD; NCAM1: Neural Cell Adhesion Molecule (CD56); NFKB1: Nuclear factor NF-kappa-B p105 subunit; NFKBIA: IκBα (nuclear factor of kappa light polypeptide gene enhancer in B-cells inhibitor, alpha; NFκB: NF-κB (nuclear factor kappa-light-chain-enhancer of activated B cells; NOV: Nephroblastoma overexpressed; NT5E: -5′-nucleotidase (CD73); OSGIN1: Oxidative stress induced growth inhibitor 1; OSMR: Oncostatin M receptor; PLG: Plasmin; PMEL: Premelanosome protein; PRKCA: Protein kinase C alpha; PRKCB: Protein kinase C beta type; RAF1: RAF proto-oncogene serine/threonine-protein kinase; RAR: Retinoic acid receptor; RMA: Robust microarray analysis; ROCK2: Rho-associated protein kinase; SERPINE1: Plasminogen activator inhibitor-1; SOCS1: Suppressor of cytokine signaling 1; SOCS3: Suppressor of cytokine signaling 3; SOX3: SRY-related HMG-box 3; SOX6: Transcription factor SOX-6; SOX9: SRY (sex determining region Y)-box 9; SPP1: Secreted phosphoprotein 1; SRC: Proto-oncogene tyrosine-protein kinase; STAT: Signal transducer and activator of transcription; STAT3: Signal transducer and activator of transcription 3; TACs: Transient amplifying cells; TGFB: Transforming growth factor, beta; TGFB1: Transforming growth factor beta 1; TGFB2: Transforming growth factor-beta 2; TGFBI: Transforming growth factor, beta-induced; TGFBR1: Transforming growth factor, beta receptor I/activin A receptor type II-like kinase; TGFBR2: Transforming growth factor, beta receptor II; THY-1: Thymocyte differentiation antigen 1; TIMP1: TIMP metallopeptidase inhibitor 1; TNFα: Tumor necrosis factor-alpha; TSP-1: Thrombospondin 1; VCAM: Vascular cell adhesion protein 1; VEGF: Vascular endothelial growth factor; VEGFA: Vascular endothelial growth factor A; VIM: Vimentin; WNT1: Proto-oncogene protein Wnt-1; WNT5A: Protein Wnt-5a.

## Competing interests

The authors declare that they have no competing interests.

## Authors’ contributions

ZV, RA and GP carried out the isolation and cultivation of cells, OKO carried out the microarray analysis, ZV, SP and OKO carried out the gene array data and IPA pathway analysis, ZV, MCM and GP overviewed and validated the gene array analysis, ZV performed the ELISA and FACS measurements, SA performed the transmission electron microscopy, ZV, MCM and GP participated in the design of the study and performed the statistical analysis. GP conceived of the study, and participated in its design and coordination. All authors read and approved the final manuscript.

## Supplementary Material

Additional file 1: Figure S1 Surface protein level analysis by FACS. Positivity of the LECSs and CECs for CD73, CD90/Thy-1, CD117/c-kit, CD146/MCAM stemness markers, CD29/Integrin β1, CD44/H-CAM cell adhesion molecules and CD47 cell viability and immunoregulatory marker, were determined by flow cytometry. CD45 was used as a negative control in these cells (Data shown are Mean ± SD; p < 0.05 *, p < 0.01 **, p < 0.001 ***; N = 6). Click here for file

Additional file 2: Figure S2 Networks generated by IPA which are related to the IL-6 signaling pathway. The colored genes appear in the studied dataset, red colored genes are up-, while green colored genes are down-regulated. The grey colored genes did not fit the cut off level. (A). 44 upstream regulators of the IL-6 signaling pathway in LESCs when grouped upon biological functions of a molecule type (B). Click here for file

Additional file 3: Figure S3 Networks generated by IPA which are related to the IL-8 mediated signaling pathway. IL-8 plays a key role in innate immunity (A) and as pro-angiogenic cytokine (B). Click here for file

Additional file 4: Figure S4 Secreted IL-6 and IL-8 levels in LESC cultures. The levels of secreted IL-6 and IL-8 as measured by ELISA in the supernatants of long term LESC cultures. (N = 21, p values were determined by student’s T test). Click here for file
