# Supplementary figures and images for: Comparison of upstream regulators in human ex vivo cultured cornea limbal epithelial stem cells and differentiated corneal epithelial cells
Source: BMC Genomics. 2013 Dec 17;14:900. doi: 10.1186/1471-2164-14-900 (PMC3880589; doi:10.1186/1471-2164-14-900)

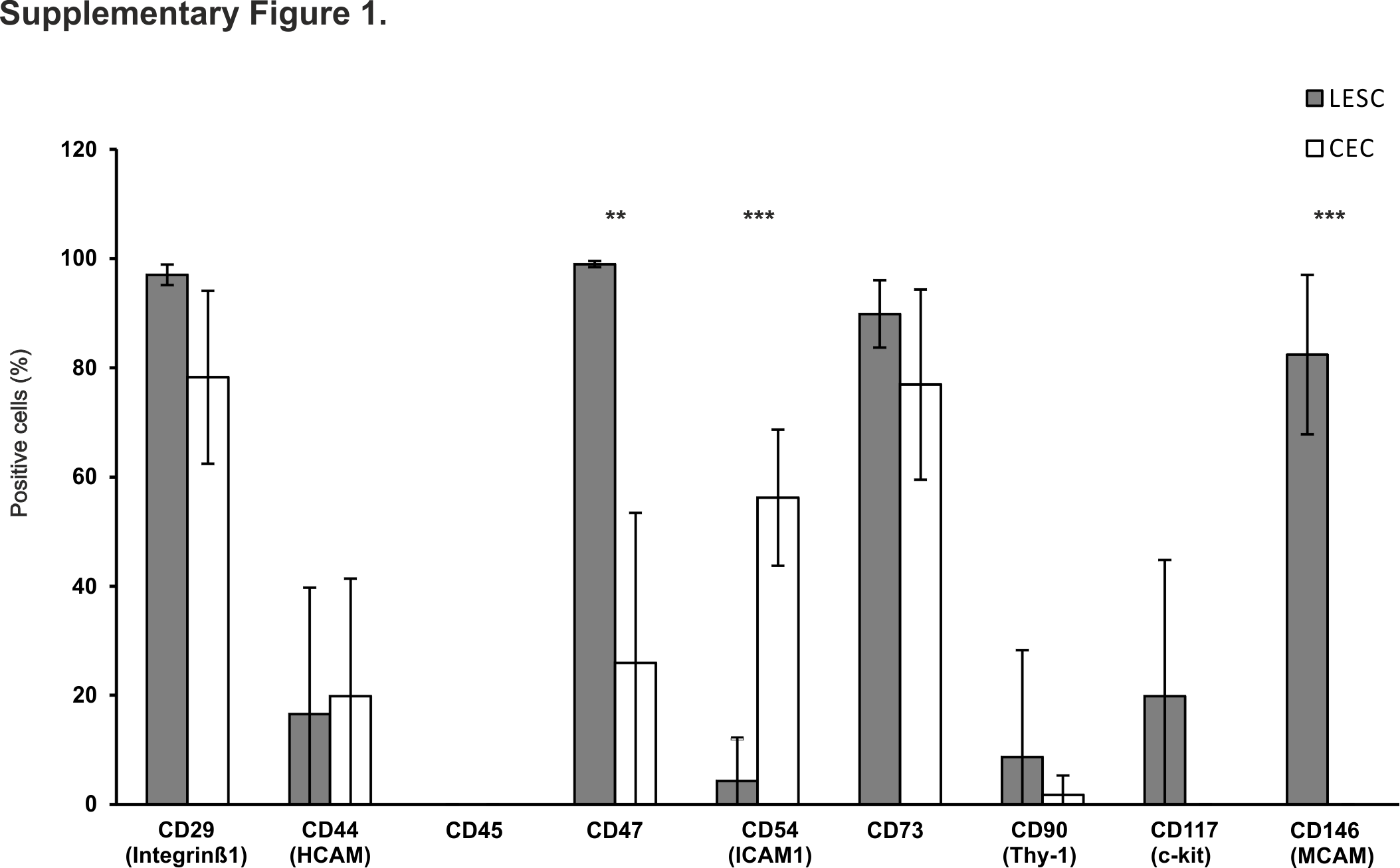

Supplement: Additional file 1: Figure S1 — Surface protein level analysis by FACS. Positivity of the LECSs and CECs for CD73, CD90/Thy-1, CD117/c-kit, CD146/MCAM stemness markers, CD29/Integrin β1, CD44/H-CAM cell adhesion molecules and CD47 cell viability and immunoregulatory marker, were determined by flow cytometry. CD45 was used as a negative control in these cells (Data shown are Mean ± SD; p < 0.05 *, p < 0.01 **, p < 0.001 ***; N = 6). [file 1471-2164-14-900-S1.tiff]

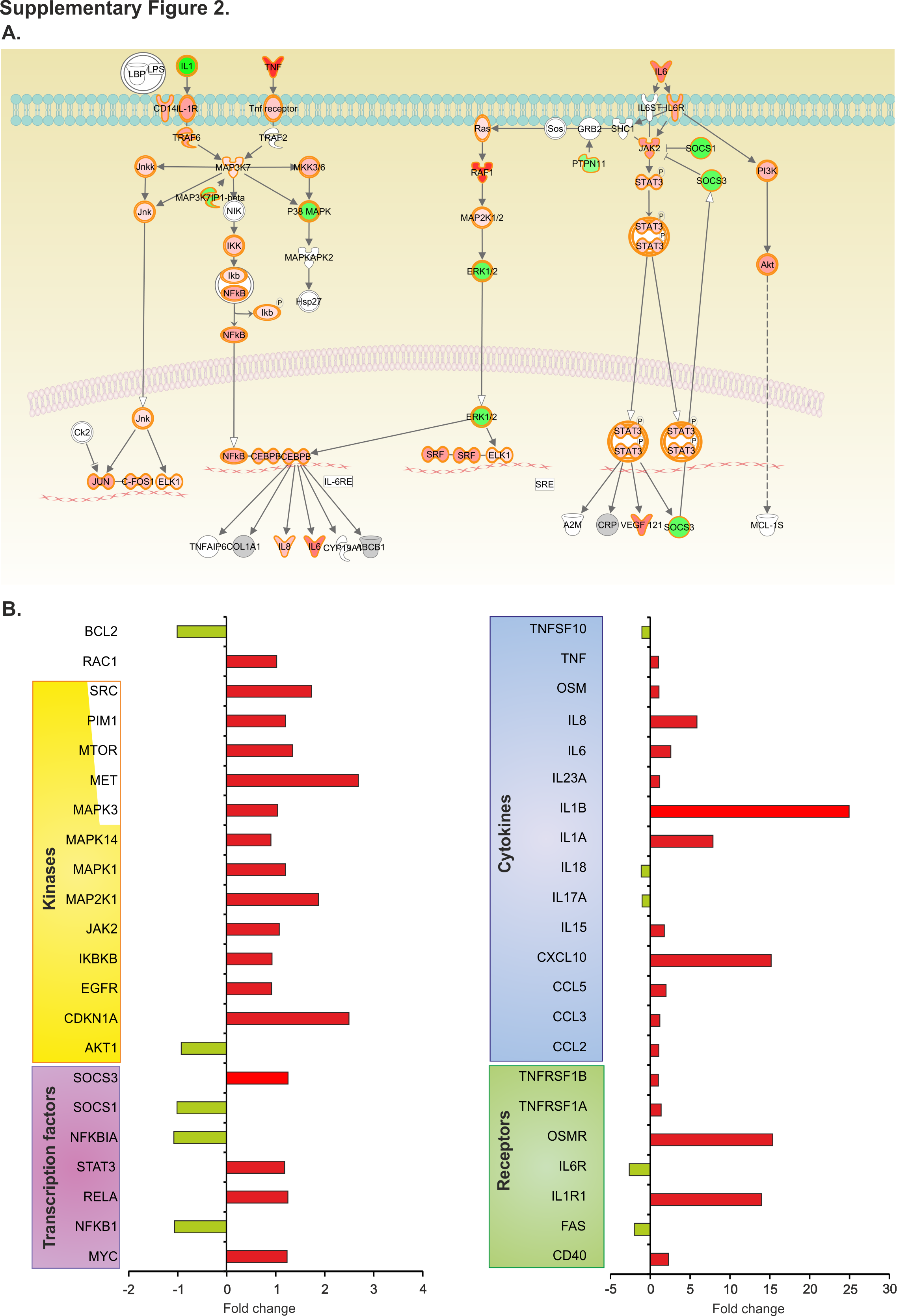

Supplement: Additional file 2: Figure S2 — Networks generated by IPA which are related to the IL-6 signaling pathway. The colored genes appear in the studied dataset, red colored genes are up-, while green colored genes are down-regulated. The grey colored genes did not fit the cut off level. (A). 44 upstream regulators of the IL-6 signaling pathway in LESCs when grouped upon biological functions of a molecule type (B). [file 1471-2164-14-900-S2.tiff]

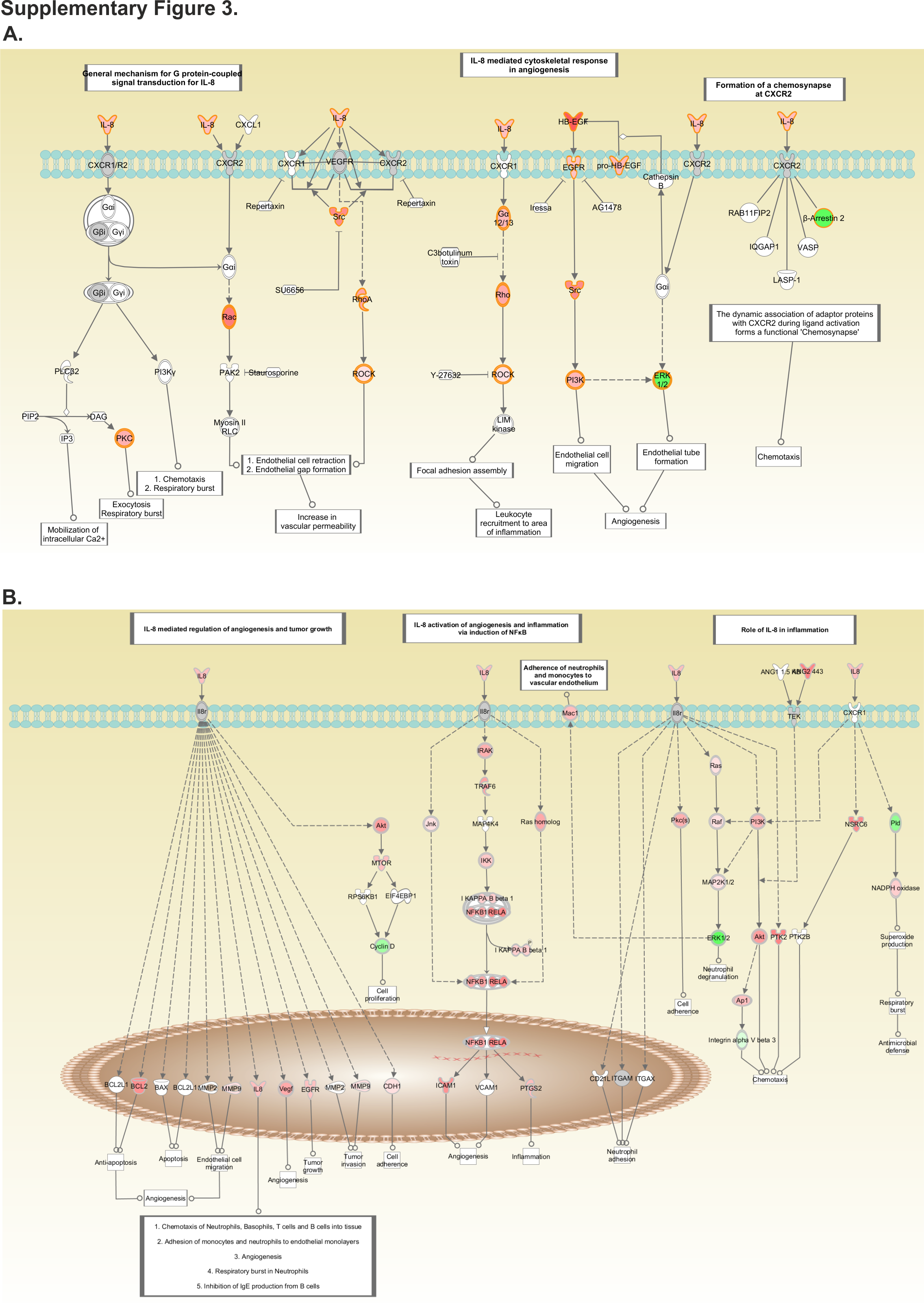

Supplement: Additional file 3: Figure S3 — Networks generated by IPA which are related to the IL-8 mediated signaling pathway. IL-8 plays a key role in innate immunity (A) and as pro-angiogenic cytokine (B). [file 1471-2164-14-900-S3.tiff]

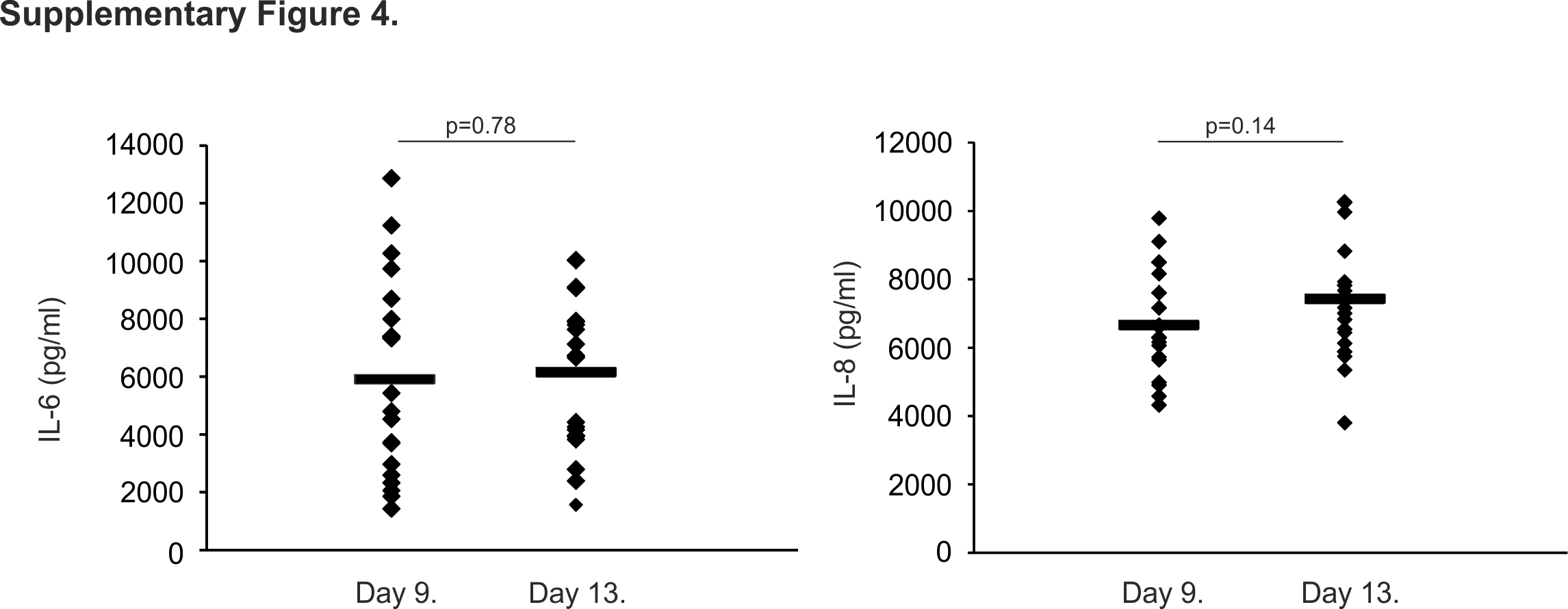

Supplement: Additional file 4: Figure S4 — Secreted IL-6 and IL-8 levels in LESC cultures. The levels of secreted IL-6 and IL-8 as measured by ELISA in the supernatants of long term LESC cultures. (N = 21, p values were determined by student’s T test). [file 1471-2164-14-900-S4.tiff]
